# Southern African HIV Clinicians Society Guideline for the clinical management of syphilis

**DOI:** 10.4102/sajhivmed.v25i1.1577

**Published:** 2024-04-30

**Authors:** Remco P.H. Peters, Jeremy S. Nel, Eitzaz Sadiq, Tendesayi Kufa, Derrick P. Smit, Gillian Sorour, Nigel Garrett, Katherine Gill, Lehlohonolo Makhakhe, Nomathemba C. Chandiwana, Neil F. Moran, Karen Cohen, Camilla Wattrus, Mahomed Yunus Moosa

**Affiliations:** 1 Research Unit, Foundation for Professional Development, East London, South Africa; 2 Department of Medical Microbiology, University of Pretoria, Pretoria, South Africa; 3 Division of Medical Microbiology, Faculty of Health Sciences, University of Cape Town, Cape Town, South Africa; 4 Division of Infectious Diseases, Department of Medicine, University of the Witwatersrand, Johannesburg, South Africa; 5 Helen Joseph Hospital, Johannesburg, South Africa; 6 Department of Neurosciences, Division of Neurology, University of the Witwatersrand, Johannesburg, South Africa; 7 Centre for HIV and STIs, National Institute for Communicable Diseases of the National Health Laboratory Services, Johannesburg, South Africa; 8 School of Public Health, Faculty of Health Sciences, University of the Witwatersrand, Johannesburg, South Africa; 9 Division of Ophthalmology, Faculty of Medicine and Health Sciences, Stellenbosch University, Cape Town, South Africa; 10 Department of Paediatrics and Child Health, Faculty of Health Sciences, University of the Witwatersrand, Johannesburg, South Africa; 11 Centre for the AIDS Programme of Research in South Africa (CAPRISA), University of KwaZulu-Natal, Durban, South Africa; 12 Department of Public Health Medicine, School of Nursing and Public Health, University of KwaZulu-Natal, Durban, South Africa; 13 Desmond Tutu Health Foundation, Cape Town, South Africa; 14 Department of Dermatology, University of the Free State, Bloemfontein, South Africa; 15 The South African Institute of Dermatology, Bloemfontein, South Africa; 16 Wits Ezintsha, Faculty of Health Sciences, University of the Witwatersrand, Johannesburg, South Africa; 17 KwaZulu-Natal Department of Health, Pietermaritzburg, South Africa; 18 Department of Obstetrics and Gynaecology, School of Clinical Medicine, College of Health Sciences, University of KwaZulu-Natal, Durban, South Africa; 19 Department of Medicine, Division of Clinical Pharmacology, University of Cape Town, Cape Town, South Africa; 20 Southern African HIV Clinicians Society (SAHCS), Johannesburg, South Africa; 21 Department of Infectious Disease, Division of Internal Medicine, Nelson R. Mandela School of Medicine, University of KwaZulu-Natal, Durban, South Africa

**Keywords:** syphilis, syphilis treatment, syphilis management, syphilis diagnosis, *Treponema pallidum*, congenital syphilis, neurosyphilis, ocular syphilis, presumptive syphilis.

## Abstract

Syphilis, ‘the great imitator’, caused by *Treponema pallidum* infection, remains a complex and multifaceted disease with a rich history of clinical diversity. This guideline aims to be a comprehensive guide for healthcare workers in Southern Africa, offering practical insights into the epidemiology, pathogenesis, clinical manifestations, diagnostic testing, therapeutic principles, and public health responses to syphilis. Although the syphilis burden has declined over the years, recent data indicate a troubling resurgence, particularly among pregnant women and neonates. This guideline highlights the diagnostic challenges posed by syphilis, stemming from the absence of a single high-sensitivity and -specificity test. While treatment with penicillin remains the cornerstone of treatment, alternative regimens may be used for specific scenarios. We highlight the importance of thorough patient follow-up and management of sex partners to ensure optimal care of syphilis cases. In the context of public health, we emphasise the need for concerted efforts to combat the increasing burden of syphilis, especially within high-risk populations, including people living with HIV.

## Introduction

‘He who knows syphilis knows medicine’ is a quote by Sir William Osler, that has remained a concept relevant for decades and highlights the complexity of syphilis. Syphilis is an ancient disease caused by *Treponema pallidum* subspecies *pallidum* (*T. pallidum*), a bacterium of the phylum Spirochaetes. Transmission most commonly occurs with sexual activity, but syphilis can also be transmitted vertically from mother to child, resulting in congenital syphilis. This occurs during pregnancy following bacteraemia, or at delivery following contact with *T. pallidum* in the birth canal.

Syphilis is known as ‘the great imitator’ due to its broad spectrum of clinical manifestations, the variety of organs that it may affect and protracted disease course. The lack of high-quality diagnostic testing and limited therapeutic options further complicate clinical management of patients with presumptive or diagnosed syphilis.

This guideline aims to support healthcare workers to recognise, diagnose and treat syphilis in the Southern African context, taking differences in resource availability into account. Specialist advice should be sought for complex cases.

## Epidemiology of syphilis in Southern Africa

**FIGURE 1:**
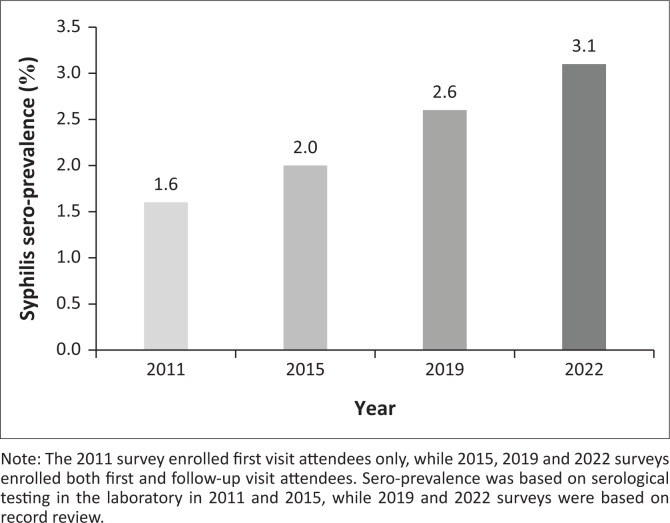
Syphilis seroprevalence estimates among pregnant women in the South African national ANC surveillance surveys over time.

In 1990, syphilis prevalence in South Africa was high, with up to 10% of the general population estimated infected;^1^ however, the introduction of syndromic management for sexually transmitted infections (STIs) has resulted in a 30% – 40% decrease in syphilis prevalence. HIV-associated mortality and antenatal care (ANC) screening contributed further to declining prevalence to less than 2% in 2015;^1,2,3^ however, data from South Africa’s national ANC HIV sentinel surveillance survey show a concerning and persistent rise in syphilis prevalence among pregnant women in recent years (Figure 1).^4,5^ The increase of syphilis in pregnancy is reflected in a concurrent rise in neonatal infections with congenital syphilis clinical case notifications and laboratory seropositivity of infants with using rapid plasma reagin (RPR) testing that have more than doubled in recent years.^6,7^ Of note, this increased prevalence has occurred against the background of global and local shortages of parenteral penicillin, resulting in treatment with regimens that are more challenging to adhere to.

Epidemiological data are limited for Southern Africa, but a higher burden of infection is reported in certain population groups, including adolescent girls and young women accessing HIV prevention services, female sex workers, men who have sex with men, transgender people, pregnant women, and people living with HIV (PLHIV).^8,9,10,11,12,13,14,15^ Syphilis prevalence is also higher in men and women presenting with genital discharge.^16,17^

## Pathogenesis of *T. pallidum* infection

**FIGURE 2:**
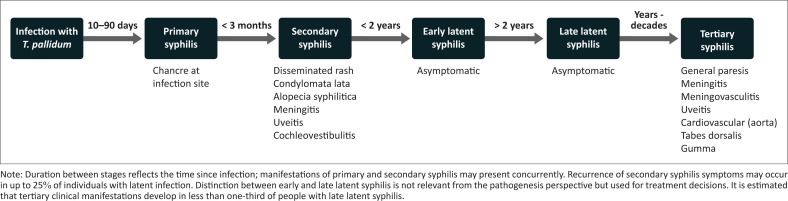
Natural history and main clinical manifestations of untreated syphilis.

Syphilis is a multistage disease with a diverse spectrum of clinical manifestations (Figure 2).^18,19^ Following exposure, infection occurs when *T. pallidum* penetrates mucous membranes or dermal micro-abrasions (e.g. of the finger), resulting in a primary ulcer (chancre) at the site of inoculation. The ulcer develops an average of 3 weeks after exposure (10–90 days) and spontaneously heals within 4–6 weeks.^18^ Bacterial dissemination occurs within hours of inoculation and during evolution of primary stage syphilis. Manifestations of secondary syphilis occur within 3 months of the initial infection and resolve spontaneously within 3 months of appearance.^18^ Primary and secondary disease may therefore present concurrently.^20,21^

If untreated or inadequately treated, the initial symptomatic stage is followed by a latent stage of asymptomatic infection. Latent infection is considered early if it occurs within 2 years after infection (different definitions are used globally) and late latent after a 2-year period. Sexual transmission is considered uncommon during late latent infection but transmission to the foetus can still occur following bacteraemia.^18^ The risk of transmission is highest in primary and secondary syphilis (as opposed to latent syphilis), but a large proportion of vertical transmissions also occur in mothers with latent syphilis as this is the more common manifestation. Untreated, approximately one-third of the individuals with late latent syphilis will develop clinical manifestations of tertiary syphilis,^19^ which may appear anytime from a few years up to several decades following initial infection.

Untreated syphilis in pregnancy can result in bacteraemia with transplacental transmission of spirochaetes to the foetus.^18^ This can occur at any stage of pregnancy and can result in various adverse outcomes such as foetal death (miscarriage or stillbirth), prematurity, intrauterine growth restriction, hydrops fetalis or infants born with congenital syphilis. The placenta is large, pale and greasy, and the umbilical cord has a classical ‘barber pole’ appearance due to necrotising funisitis: an inflammation of the umbilical cord characterised by spiral stripes of red and blue discolouration.^22,23^

## Clinical manifestations of syphilis

### Dermatological manifestations

**IMAGE 1:**
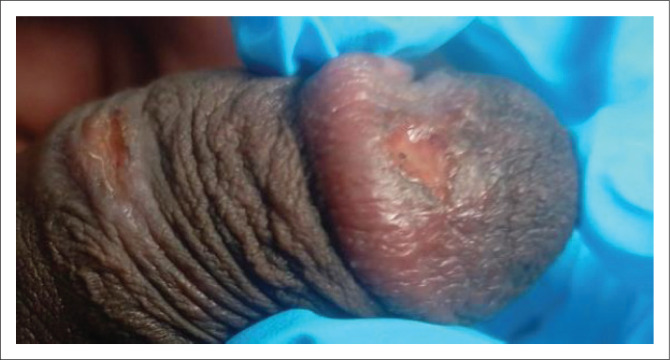
Genital ulcer in a man.

**IMAGE 2:**
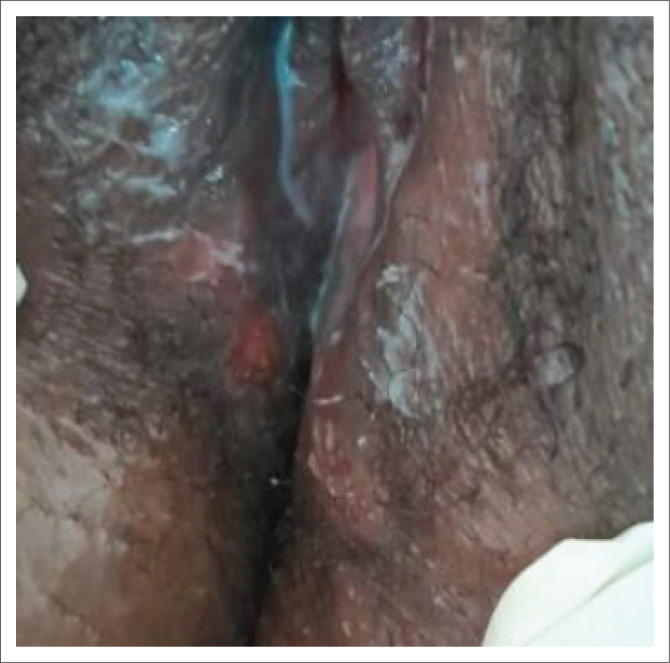
Genital ulcer in a woman.

Primary syphilis usually presents as a papule at the point of entry that breaks down into an ulcer (chancre). The ulcer is classically solitary, firm, indurated, 0.5 cm – 2 cm in diameter and is often painless (Images 1 and 2).^24^ Although this is considered the typical syphilis presentation, atypical manifestations are commonly observed, including nonindurated lesions with irregular borders, multiple, confluent and/or painful lesions.^18,25^ The glans penis, vulva and cervix are the most common locations for the ulcer, followed by the mouth and rectum. A particular presentation is a ‘kissing ulcer’, which may be caused by various genital infections, which refers to an ulcer that occurs in a fold of the skin or mucous membrane and forms symmetrical lesions on either side.^26^ Chancres of the finger(s) are sometimes observed.^27^ Importantly, the appearance and presentation of clinical ulcer(s) has poor diagnostic accuracy for predicting aetiology and should not be used to make treatment decisions.^28,29^

Up to 30% of syphilis infections are acquired through condomless oral sex, making the mouth an important anatomic location for physical examination.^18,24^ The two main clinical phenotypes are (reddish) papules and ulcerative lesions as a manifestation of primary syphilis, and greyish-white mucous patches as part of secondary syphilis; these lesions are usually located at the (upper) lip and tongue, followed by the palate and buccal mucosa.^30,31,32^

**IMAGE 3:**
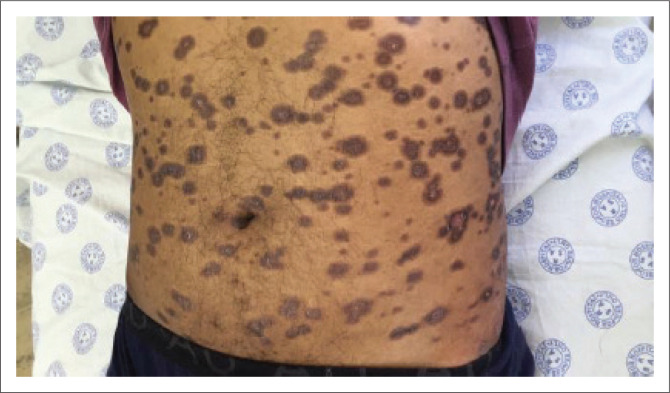
Generalised mucocutaneous rash.

**IMAGE 4:**
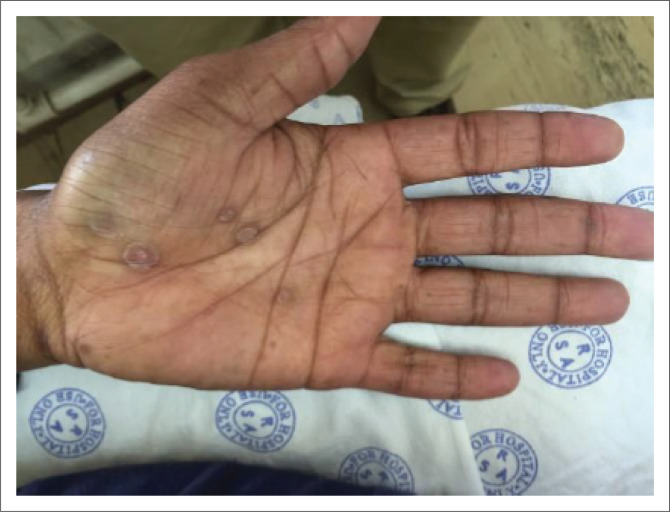
Palmar rash.

**IMAGE 5:**
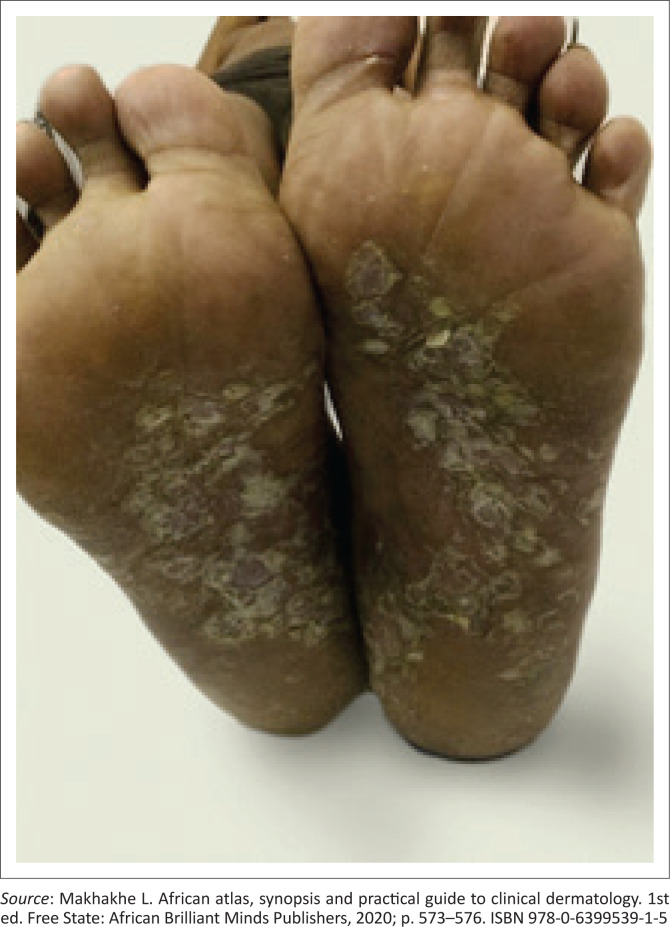
Rash on soles of feet.

**IMAGE 6:**
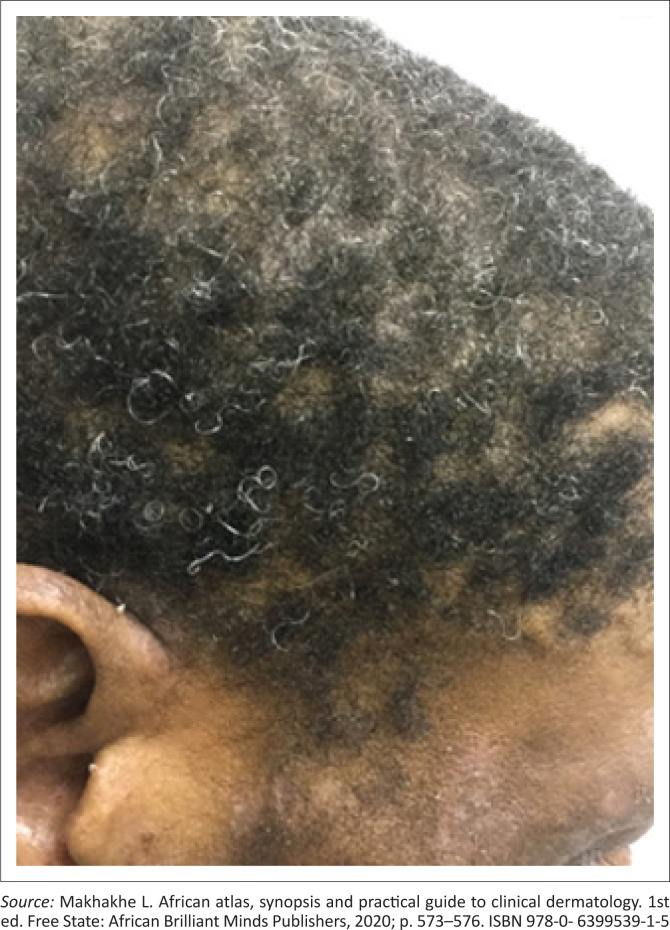
Alopecia in a moth-eaten pattern.

**IMAGE 7:**
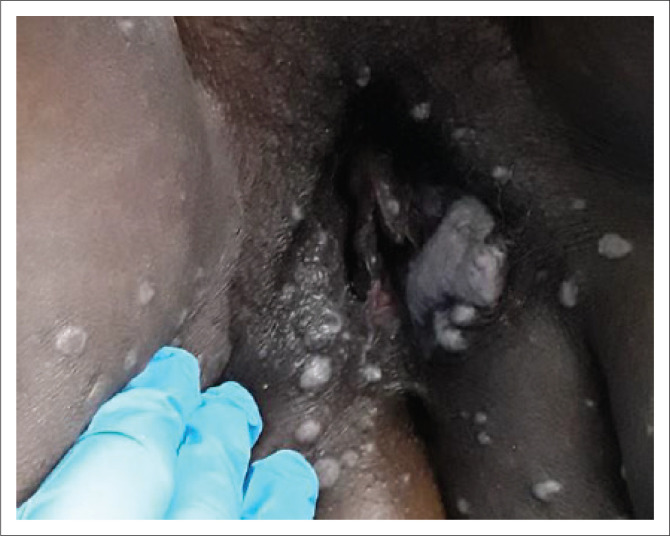
Condylomata lata.

A common manifestation of secondary syphilis is a non-specific generalised mucocutaneous rash (Image 3), often combined with systemic symptoms such as malaise, muscle aches and generalised lymphadenopathy. The skin lesions can range from a mild morbilliform rash to widespread annular plaques, ham-like papules, macules, and ulcers with marked crusting and scaling. The rash is frequently found on the palms of the hands (Image 4) and the soles of the feet (Image 5). In rare cases, lesions may become necrotic (malignant syphilis). Infection of the hair follicles resulting in alopecia of the scalp is present in approximately 10% of patients, and typically presents in a moth-eaten pattern (Image 6).^33^ Up to 10% of patients develop condylomata lata (Image 7).^34,35^ These lesions are mostly skin-coloured and are typically smooth, soft and flat, occurring in warm and moist areas such as the anogenital region, toe webs and oral cavity.^34,35^ They may vary in size and shape and are highly infectious.^34,35^

Dermatological manifestations of primary and secondary syphilis frequently go unnoticed or are ignored by the patient because they may occur in a place where the patient does not see them (e.g. vagina/cervix or rectum), does not recognise them (e.g. mouth), or may appear mild and transient (e.g. as single painless papule). Both primary and secondary stages usually self-resolve, even in the absence of antibiotic therapy; however, the secondary manifestations may recur in up to 25% of individuals with latent infection.^18,36^

### Neurological manifestations

Neurosyphilis may present within several weeks after initial infection; contrary to the common misconception that neurological manifestations of syphilis only appear late in the disease process.^18^ Syphilitic meningitis is the most common presentation and manifests as headache, neck stiffness, photophobia, confusion, and/or seizures. Cranial nerve palsies, ocular and auditory pathology may also be noted at this stage.^37,38^ Other less common neurological manifestations that may occur from early on include meningovascular syphilis (presenting with focal neurological deficits due to strokes) and meningomyelitis causing slowly progressive spastic paraparesis.^37,38^

**IMAGE 8:**
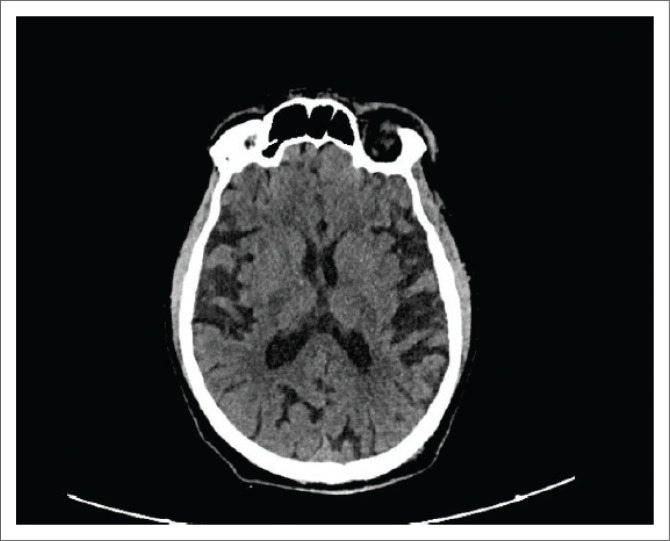
CT scan showing generalised cerebral atrophy.

Late neurosyphilis typically presents 10–30 years post infection, usually with a syndrome of progressive dementia and neuropsychiatric manifestations ranging from subtle personality changes to depression and florid psychosis. The neuropsychiatric manifestations of late syphilis are also called ‘general paresis’.^37,38^ Tremor, dysarthria and ataxia may also be noted, and patients may become bedridden with advanced disease. Tabes dorsalis is a progressive spinal cord disorder presenting with areflexia, sensory ataxia, incontinence and lancinating pains in the abdomen and legs. It has become very rare in the antibiotic era, as have syphilitic gummata in the central nervous system.^39^ Image 8 shows generalised cerebral atrophy seen in advanced disease.

HIV infection is associated with an increased likelihood of developing neurosyphilis, particularly in patients with significant immunosuppression.^40^ The clinical manifestations may be more severe and atypical in PLHIV, which should prompt a lower threshold to investigate for neurosyphilis in patients presenting with neurological symptoms.^40^

### Ocular manifestations

*T. pallidum* infection may affect almost every structure of the eye and usually presents during the secondary and tertiary stages.^41^ Ocular syphilis is an important cause of ocular inflammation and may manifest in different ways.^42^ Posterior uveitis and panuveitis are the most common forms and these occur more commonly in PLHIV. Other ocular inflammatory manifestations are anterior uveitis, vitritis, optic neuritis, choroiditis, retinitis, or vasculitis. Patients commonly complain of eye pain, sensitivity to bright light (photophobia), loss of vision, red eye, or floaters in their field of vision.

Ocular syphilis is more common in PLHIV and there is a stronger association between ocular and neurosyphilis when compared to those without HIV.^43,44^ Any patient who presents with signs of ocular inflammation should be tested for syphilis, especially those with posterior uveitis and panuveitis.

### Manifestations in the newborn

Diagnosis of congenital syphilis is challenging and can be easily missed as it is asymptomatic in most newborns (60% – 90%) at birth.^45^ If the exposed newborn is not treated, symptoms usually develop within weeks to months. Congenital syphilis is divided into early (< 2 years of age) and late (> 2 years of age) disease (Table 1).^46,47^

**TABLE 1:** Most common clinical manifestations of congenital syphilis by stage of *T. pallidum* infection.

Early congenital syphilis (< 2 years)	Late congenital syphilis (> 2 years)
Prematurity	Interstitial keratitis†
Growth restriction	Sensorineural hearing loss
Nasal discharge (‘Snuffles’)	Hutchinson’s teeth‡
Mucocutaneous rash (incl. desquamation)	Rhagades§ (nares, lip and anus)
Hepatomegaly	Mulberry molars¶
Splenomegaly	Bone abnormalities (skull, maxilla, palate, nose, shin)
Jaundice	Ocular (uveitis, optic atrophy)
Pallor, petechiae	Developmental delays
Condylomata lata	-
Oedema (non-immune hydrops fetalis)	-
Long bone changes, osteochondritis, periostitis	-
Acute meningitis, seizures	-
Ocular (chorioretinitis, uveitis, cataract, glaucoma)	-

Note: Manifestations of primary and secondary congenital syphilis may present concurrently.

† , Non-ulcerating inflammation of the corneal stroma causing decreased vision, photophobia and pain. Cornea appears hazy with conjunctival injection.

‡ , Peg-shaped, notched upper incisors.

§ , Linear fissures or cracks.

¶ , Multiple rounded rudimentary enamel cusps on the permanent first molars.

**IMAGE 9:**
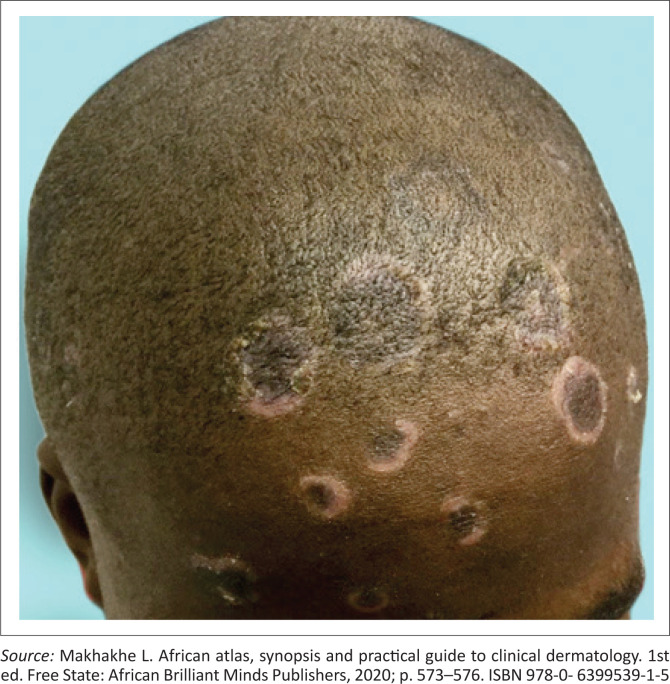
Annular rash in secondary syphilis.

Early signs are variable, can affect any organ system and resemble those seen in secondary syphilis in adults. Three of the commonest clinical signs for early congenital syphilis at a tertiary hospital in South Africa were respiratory distress, hepatosplenomegaly and petechiae.^48^ A thick or bloody nasal discharge (‘snuffles’) is one of the earliest signs of congenital syphilis and usually occurs 1–2 weeks before the onset of a maculopapular rash involving the hands and feet, which classically desquamates. Other dermatological manifestations include a bullous rash, an annular rash (Image 9), patches on the oral mucosa and condylomata lata.^23,46,47^ Other symptoms include painful osteochondritis resulting in irritability and pseudoparalysis of the involved limb (‘Pseudoparalysis of Parrot’), destruction of the medial portion of the proximal tibial metaphysis (Wimberger’s sign), ocular inflammation (uveitis and chorioretinitis with secondary cataract or glaucoma), hepatosplenomegaly with possible jaundice resulting from extramedullary haematopoiesis and other reticuloendothelial signs such as haemolytic coombs-negative anaemia and thrombocytopaenia.

The signs of late congenital syphilis are usually due to chronic inflammation of bone, teeth and the central nervous system and are now less commonly seen due to syphilis screening in pregnancy and treatment of exposed infants. Signs include Hutchinson’s triad (peg-shaped, notched upper incisors; interstitial keratitis; and eighth nerve deafness), ‘saddle nose’ (collapsed nasal root due to syphilitic rhinitis destroying adjacent bone and cartilage), a defect in the hard palate and rhagades (linear scars from previous mucocutaneous fissures of the mouth, anus and genitalia), and ‘sabre shin’.^46,47,49^ Ocular manifestations (uveitis, optic atrophy) and neurodevelopmental delay may also occur.^47,49^

## Diagnostic testing for syphilis

### Principles of diagnostic testing

Laboratory diagnosis of syphilis can be challenging due to the biology of *T. pallidum*. Direct observation of the bacteria through darkfield microscopy is generally not available, and culture requires highly specialised facilities.

Detection of *T. pallidum* DNA through nucleic acid amplification test (NAAT) has a sensitivity and specificity of 80% – 90% for primary syphilis assuming adequate sampling of ulcer edge is performed.^50^ A negative NAAT therefore does not rule out infection, but a positive test confirms the diagnosis.

Serological diagnosis of syphilis requires positive results from both a treponemal and non-treponemal test. Treponemal tests that are commonly available include enzyme immunoassay (EIA) or chemiluminescent immunoassay (CLIA) to detect IgM and IgG immunoglobulins and the treponemal antibody assays (*T. pallidum* haemagglutination assay [TPHA], *T. pallidum* particle agglutination assay [TPPA] or fluorescent treponemal antibody absorption [FTA-ABS] test). Non-treponemal tests are the RPR and venereal disease research laboratory (VDRL) test. Sensitivity of the treponemal tests is 70% – 90% in early disease and the window period of the test exceeds the incubation period of the disease;^51^ meaning that negative serology does not exclude syphilis in (early) symptomatic individuals.^29^

Clinical, treatment and travel history should be taken to interpret the serological profile in any patient as this may reflect various scenarios. For example, a positive treponemal and negative non-treponemal test may indicate early, late latent, and previously treated syphilis (and symptoms may be due to another aetiology in the latter case).

Treponemal tests have a higher sensitivity than non-treponemal tests. Therefore, most laboratories in Southern Africa have implemented the so-called ‘reverse algorithm’ with detection of IgM and IgG immunoglobins as the first step, which is an automated test that has a shorter window period than the manual TPHA/TPPA. If positive, this is then followed by a manual reflex RPR or VDRL.

Syphilis rapid diagnostic tests (RDTs) have recently been introduced for syphilis point-of-care testing, especially in ANC services. These RDTs detect *T. pallidum* antibodies and are available as single test or combined with an HIV test. Sensitivity and specificity of these RDTs are comparable to laboratory-based treponemal assays for screening purposes. However, the lower sensitivity of RDTs in primary syphilis means that a negative test does not exclude syphilis.^52,53^ As with the reverse algorithm, described above, a positive RDT screening test should immediately be followed by an RPR test to confirm current infection and establish a baseline RPR titre.

### Diagnostic approaches

Syphilis diagnostic test recommendations are based on the clinical presentation, and access and availability to resources, and should be part of a comprehensive diagnostic work-up (Table 2). Serological testing should be performed in all patients suspected of syphilis, with the objective of establishing the diagnosis and to document pre-treatment RPR titre as a baseline for subsequent monitoring. Due to the window period, it is reasonable to repeat serology after 1 week in case of high clinical suspicion of primary syphilis and negative result of the first test.

**TABLE 2:** Diagnostic tests recommended in a work-up for presumptive syphilis.

Clinical manifestation	NAAT for *T. pallidum*	Antibody testing	Comment
Genital ulcer	Swab	Serum	Tests may be negative in early infection
Oral lesions	Swab	Serum	-
Maculopapular rash	-	Serum	Biopsy not recommended
Condylomata lata	Swab	Serum	Biopsy not recommended
Alopecia	-	Serum	Trichoscopy not recommended
Neurological manifestations	CSF	CSF + serum	Elevated CSF cellularity and protein levels are supportive
Ocular manifestations	Aqueous humour	Aqueous humour + serum	Vitreous fluid may also be used

NAAT, nucleic acid amplification test; CSF, cerebrospinal fluid.

Resources permitting, a swab of a genital ulcer, presumptive condylomata lata or oral lesions suspicious of syphilis should be sent for NAAT to detect *T. pallidum* DNA, as well as detection of herpes simplex virus (HSV)-1 and HSV-2 DNA as the main differential diagnosis. A positive NAAT for HSV will confirm the diagnosis, while a negative NAAT makes HSV unlikely but does not rule out syphilis.^29^

#### Neurosyphilis

**FIGURE 3:**
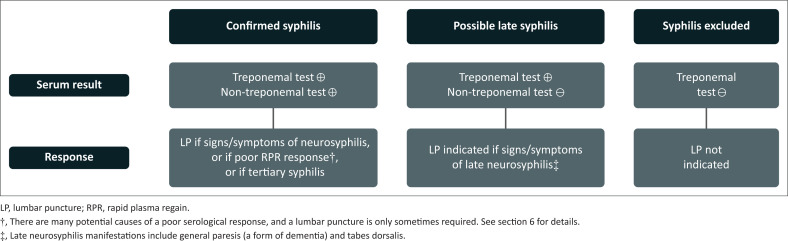
When to perform a lumbar puncture in patients with confirmed or suspected syphilis.

In a patient with *confirmed syphilis* (i.e. serum treponemal and non-treponemal tests both positive), a lumbar puncture is indicated in the following scenarios (Figure 3):

- If there are signs and/or symptoms of neurosyphilis.
- If the serum RPR titre fails to decline appropriately despite appropriate treatment, assuming reinfection is thought to be unlikely (see section on therapeutic principles for details). Of note, asymptomatic neurosyphilis is one reason for treatment failure.
- If there is evidence of tertiary syphilis, for example cardiovascular, ocular or peripheral neurological manifestations, because up to 30% – 40% of these cases can have asymptomatic neurosyphilis.

Empiric treatment may be considered for patients with a high index of suspicion for neurosyphilis in whom a lumbar puncture is either contraindicated or technically not feasible.

By contrast, for patients with a *negative* serum non-treponemal test (i.e. in whom active syphilis is in doubt):

- A negative serum treponemal test effectively rules out neurosyphilis without the need for a lumbar puncture.
- A positive serum treponemal test may still indicate neurosyphilis in rare patients with late tertiary neurosyphilis, in whom serum non-treponemal tests can sometimes revert to negative. Thus, in patients presenting with signs compatible with general paresis (a form of dementia), tabes dorsalis, or a central nervous system (CNS) syphilitic gumma, a lumbar puncture should be performed. For most other patients with a negative serum treponemal test, a lumbar puncture is not indicated unless the pre-test suspicion for neurosyphilis is very high.

Cerebrospinal fluid (CSF) treponemal tests (e.g. FTA-ABS) have a high sensitivity and can help exclude neurosyphilis, but specificity is low; therefore, a positive test requires further evaluation. CSF non-treponemal tests (e.g. VDRL) may help to confirm the diagnosis if they are positive, but they are not sensitive, and so a negative non-treponemal test is of limited value.^54^

Cerebrospinal fluid cellularity and protein concentration should also be assessed, as lymphocytic pleocytosis and elevated protein levels suggest (but absence does not exclude) the diagnosis of neurosyphilis.^54,55^

#### Ocular syphilis

Ocular syphilis is diagnosed if a patient has ocular inflammation compatible with syphilis upon physical examination combined with a positive serology result. Syphilis serology is usually positive in ocular disease as this manifestation generally occurs during the second and third stages of disease. *T. pallidum* antibody and DNA testing of aqueous humour, if obtained, could help confirm the diagnosis, or be part of testing for a differential diagnosis when patients present with ocular symptoms and signs.^56^ A lumbar puncture is not routinely indicated to diagnose ocular syphilis as it does not have therapeutic consequences.

#### Congenital syphilis

**FIGURE 4:**
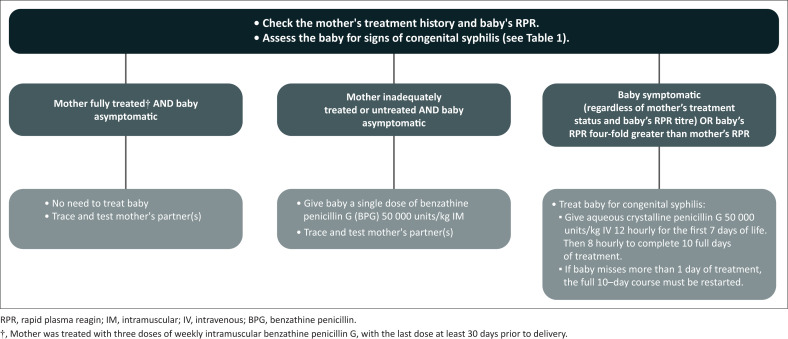
Management of the syphilis-exposed infant.

Laboratory diagnosis of congenital syphilis can be challenging because maternal antibodies cross the placenta during pregnancy. Treponemal tests in neonates are therefore not usually recommended because they are difficult to interpret, as maternal antibodies may persist for more than 15 months, and a positive result will merely reflect the mother’s syphilis status. Non-treponemal tests (RPR or VDRL) are preferred as first-line testing when there is a suspicion of syphilis, but they should always be interpreted in the context of the maternal titre, treatment history and a thorough physical examination of the neonate for signs of congenital syphilis. The RPR titre of the infant should be compared to that of the mother. An RPR titre four-fold greater than the mother’s titre is highly suggestive of congenital syphilis but a titre lower than the mother’s titre can occur, and if there are signs of syphilis, the infant should be treated irrespectively (Figure 4). If the placenta is available, it should be sent for histology.

Further investigations that could aid diagnosis include long bone and chest x-rays, a full blood count (looking for anaemia and thrombocytopaenia) and liver function tests (raised alanine transaminase/aspartate transaminase and/or bilirubin). A lumbar puncture with a VDRL requested on the CSF should only be done if there are CNS signs in the infant. Ophthalmology referral and audiology screening may be necessary if ocular or oto-syphilis are clinically suspected. Serological confirmation is not required to make a clinical diagnosis and initiate treatment when clinical signs of congenital syphilis are present and there is a history that the mother had untreated or incompletely treated syphilis during the pregnancy.

## Therapeutic principles

### Antibiotic regimen of choice

The drug of choice for all forms of syphilis remains penicillin G (Table 3). Despite over 80 years of use, *T. pallidum* appears to have never developed resistance to penicillin.^57^ Shorter durations of treatment are sufficient in early syphilis, but longer courses are generally recommended for late latent or tertiary syphilis. Evidence for the recommendation for two additional doses in late latent infection is limited and based on the theoretical concern that *T. pallidum* organisms may divide more slowly in later disease stages, thereby requiring prolonged therapy for eradication.^58^

**TABLE 3:** First-line and alternative regimens recommended for treatment of syphilis.

Type	First-line regimen	Alternative regimen
Primary syphilis (e.g. genital ulcer)	Benzathine penicillin G 2.4 million units IM single dose.	Doxycycline 100 mg twice daily for 14 days†.
Secondary syphilis (e.g. mucocutaneous rash)	Benzathine penicillin G 2.4 million units IM single dose.	Doxycycline 100 mg twice daily for 14 days†.
Early latent (< 2 years) syphilis (asymptomatic)	Benzathine penicillin G 2.4 IM million units single dose.	Doxycycline 100 mg twice daily for 14 days†.
Late latent syphilis (> 2 years) (asymptomatic)	Benzathine penicillin G 2.4 IM million units three doses weekly.	Doxycycline 100 mg twice daily for 28 days†.
Neurosyphilis (e.g. meningitis)	Aqueous Penicillin G 3–4 million units IV 4-hourly (or 18–24 million units per day continuous IV infusion) 10–14 days.	Ceftriaxone 2 g IV daily for 10–14 days. Procaine penicillin G 2.4 million units IM daily AND Probenecid 500 mg 6-hourly for 10–14 days.
Ocular syphilis (e.g. poster uveitis)		
Pregnant women	Benzathine penicillin G 2.4 IM million units three doses weekly‡.	Consult specialist.
Congenital syphilis	If baby is asymptomatic: ■ if mother fully treated§: No treatment is required ■ if mother inadequately treated or untreated: one dose of IM Benzathine Penicillin 50 000 U/kg. If baby has clinical signs of congenital syphilis: ■ *if newborn (< 1 month):* Aqueous Penicillin G 100 000–150 000 units/kg body weight per day, administered as 50 000 units/kg body weight per dose IV every 12h during first 7 days of life and every 8h thereafter for a total of 10 days ■ *if infant/child (> 1 month)*: Aqueous Penicillin G 200 000–300 000 units/kg body weight per day, administered as 50 000 units/kg body weight per dose IV every 4–6 h for 10 days.	If baby has clinical signs of congenital syphilis: cefotaxime for 10 days with appropriate dosing and frequency according to weight, postnatal age, and gestational age.

IM, intramuscular; IV, intravenous

† , Avoid in pregnancy.

‡ , Treatment for syphilis in pregnancy is usually based on a positive screening result. The duration and stage of infection is usually unknown, therefore three doses of Benzathine Penicillin G is recommended to ensure adequate treatment of the foetus.

§ , Three weekly doses of bicillin with the last dose > 30 days before delivery.

Benzathine penicillin G (BPG) is the recommended drug of choice for most syphilis manifestations, despite a paucity of clinical trial data, but based on decades of experience.^59^ This depot formulation is given intramuscularly, providing therapeutic drug levels for several weeks following each dose. Benzathine penicillin G works as long-acting depot as it is slowly hydrolysed to penicillin G, which is short-acting and should only be used for intravenous treatment of syphilis.^60^ Treatment with BPG is highly effective, with treatment success reported in 90% – 100% of cases based on serological response.^59,61^ Treatment failure, based on serological non-response, may be more likely in PLHIV; however, there is insufficient evidence to warrant any change to the treatment regimen.^62,63^ Multiple-dose treatment does not have clinical benefit over single dose treatment for early syphilis, regardless of HIV or pregnancy status.^61,63,64^ Pregnant women and PLHIV should therefore be treated with the recommended penicillin regimen for their stage of infection.^58^ In pregnancy, however, syphilis treatment is usually administered in response to positive screening serology, rather than in response to a diagnosis based on clinical signs or symptoms. Therefore, the stage of syphilis is not usually known by the healthcare worker making the diagnosis. For this reason, it remains common practice when treating syphilis in pregnancy in South Africa for three doses of BPG, given at weekly intervals, to be given to cover any stage of syphilis. Due to the devastating effects of congenital syphilis, the tendency is to accept overtreatment of many in order to prevent the undertreatment of a few.

Intramuscular BPG does not adequately penetrate the CNS; therefore, intravenous aqueous penicillin G is recommended for the treatment of neurosyphilis, otosyphilis and ocular syphilis.^58^ Recommended treatment duration internationally is 10–14 days. In the absence of clear evidence to determine the appropriate treatment duration, and taking in-patient bed pressure into account, it is reasonable to treat for a minimum of 10 days, and continue for up to 14 days, at the healthcare worker’s discretion (e.g. treating for longer in patients with more severe clinical presentation or a slower clinical response).

Treating for congenital syphilis is dependent on the maternal treatment status and the infant’s clinical symptoms (Figure 4). If the mother and partner(s) have been fully treated for syphilis (3 weekly doses of bicillin with the last dose > 30 days before delivery) and the baby is asymptomatic, then there is no need to treat the baby. If the mother and partner(s) have not been fully treated (i.e. have not received three doses of BPG 2.4 million IU at least 1 month before the date of delivery) and the infant is asymptomatic, the infant should receive a single dose of BPG 50 000 units/kg IM.^65^ Symptomatic infants should be admitted to hospital to receive intravenous aqueous penicillin G for 10 days.^58,65^ The dose frequency changes with the age of the infant: if the infant is less than 7 days old, they should receive 50 000 units/kg 12-hourly intravenously and then 50 000 units/kg 8-hourly from day 8 of life onwards.

All symptomatic infants with a RPR titre >1:8 should be followed up three-monthly after discharge until their RPR titre decreases at least four-fold or becomes negative. In addition, they should undergo neurodevelopmental screening at each visit.

### Alternative antibiotic options

Alternative regimens may be necessary when penicillin G is contraindicated or unavailable (Table 3). A common reason to avoid penicillin formulations is concern of penicillin allergy. However, true penicillin allergy is uncommon in our region, therefore the majority of patients who report having a penicillin allergy are actually not at high risk of a severe allergic reaction.^66^ Importantly, alternative therapeutic options are available in most cases. It is therefore important to take a good history and work-up of anyone reporting penicillin allergy; penicillin desensitisation before syphilis treatment can be attempted in those with a confirmed severe allergy, although this is generally only necessary for cases of neurosyphilis and syphilis in pregnancy, where the efficacy of non-beta-lactam drug options is not well-established.

Doxycycline is the most important alternative to BPG, with similar efficacy reported in several observational studies of non-neurological syphilis.^58^ Disadvantages of doxycycline include the need to take treatment for 2 weeks in early syphilis, or 4 weeks in late latent syphilis), side-effects, adherence challenges, and concerns about teratogenicity in pregnancy. Nevertheless, given the global shortage of BPG,^67^ doxycycline is currently widely used to treat non-neurological syphilis, including in Southern Africa. Doxycycline is not recommended for neurosyphilis as efficacy has not been established.^68^

Oral azithromycin has been used for non-neurological syphilis. However, due to emerging macrolide resistance of *T. pallidum* worldwide, with almost 50% of strains reported resistant in a recent study from South Africa,^57,69^ this drug should not be used unless all other treatment options are unavailable or contraindicated.

The cephalosporins are the most commonly used alternative treatment class and these drugs might be used even with true penicillin allergy as the rate of cross-reaction to cephalosporins is very low.^70,71^ Several trials are currently underway to document efficacy of cephalosporins for different stages of syphilis.^72,73^ In case of neurosyphilis, based on observational studies, ceftriaxone or the combination of procaine penicillin G with probenecid are the main alternative therapeutic options.^74,75^ Recent observational data suggest similar efficacy in neurosyphilis between ceftriaxone and intravenous penicillin G, although there is much less experience with the former.^74,76,77^ A recently completed trial shows that linezolid 600 mg orally for 5 days should not be used for active syphilis.^78^

Infants requiring full treatment for congenital syphilis should receive 10 days of intravenous cefotaxime if parenteral penicillin is unavailable, but these infants require close clinical and serological follow-up to ensure treatment has been effective.

### Patients who fail to complete treatment

There are no evidence-based recommendations for the management of patients who fail to complete their treatment regimen for late latent syphilis. Based on the biology of *T. pallidum* infection, unclear evidence for a three-dose regimen, and the pharmacological characteristics of BPG, the following recommendations may be reasonable: if the second or third weekly dose of BPG is delayed, but given within 3 weeks of the previous dose, this can be regarded as adequate treatment. In the case of a longer interruption (more than 3 weeks) between doses, the full treatment course should be restarted. If a pre-treatment RPR titre is available, and patient follow-up can be guaranteed, RPR may be repeated to guide further treatment decisions for interruptions of more than 6 months.

There is no evidence to guide treatment decisions when a doxycycline course for syphilis treatment is interrupted. Dependent on the duration of interruption and adherence level, it is at the healthcare worker’s discretion to restart treatment (in which case BPG is also an option) or to monitor RPR for further treatment decisions.

### Jarisch-Herxheimer reaction

It is essential to counsel each patient treated for syphilis about the Jarisch-Herxheimer reaction upon treatment initiation. This reaction is an adverse event syndrome that can occur within 24 h of starting treatment for syphilis and is thought to be a cytokine-driven phenomenon caused by the release of spirochetal lipoproteins.^79,80^ Typical symptoms include fever, myalgia, headache, skin flushing, exacerbation of rash, and/or mild hypotension.^79^ This syndrome occurs in 10% – 35% of treated syphilis cases, with risk factors including early syphilis, a higher RPR titre, and first (rather than repeat) syphilis treatment with penicillin.^80^ There is no proven way to prevent the syndrome, and steroid prophylaxis is not recommended, but treatment with antipyretics may help reduce symptoms.

### Corticosteroids

In general, there is no role for steroids in the treatment of syphilis or prevention of a Jarisch-Herxheimer reaction. The only exception is in the case of ocular syphilis where corticosteroids, both topically and systemically, play an adjunctive role in treatment.^81^ Topical corticosteroid drops, such as dexamethasone 0.1% or prednisolone 1.0% drops, may be used for anterior uveitis and interstitial keratitis. Systemic corticosteroids, usually oral prednisone, may be indicated for syphilitic scleritis, vitritis and posterior uveitis, while intravenous methylprednisolone, followed by oral prednisone, may be used for optic neuritis caused by syphilis.

### Treatment of sexual partners

Sexual transmission of *T. pallidum* occurs predominantly during the early stages of syphilis and becomes uncommon during late latent infection.^18^ All recent (< 3 months) sexual partners of the index patient should be counselled and offered treatment for early syphilis, and any further/new partners traced. Diagnostic testing may be performed to confirm the serological status and allow for possible follow-up. However, treatment should not be withheld in case of a negative test result, because syphilis cannot be excluded reliably due to the window phase.

### Case notifications

Adult syphilis is not routinely reported in Southern Africa and only included in facility data for pregnant women. However, congenital syphilis is a category 2 notifiable medical condition in South Africa, meaning that all cases must be reported through a written or electronic notification within 7 days of clinical or laboratory diagnosis, but preferably as soon as possible following diagnosis. It is important for all healthcare workers to be aware of the definition of congenital syphilis (which includes any stillbirth due to syphilis infection) for notification purposes. Further information including the case notification and case investigation forms can be found on the National Institute for Communicable Diseases website: https://www.nicd.ac.za/diseases-a-z-index/congenital-syphillis/.

## Follow-up of patients treated for syphilis

Follow-up of patients treated for syphilis is important to determine the treatment response, especially if alternative regimens are used, and to identify repeat infection early. A history of treatment completion and partner management should be obtained. All patients should be followed up with serial RPR titres to assess treatment response. If there has been a delay between drawing the specimen for the RPR test and treatment initiation, a new baseline RPR titre should ideally be taken at the same time as treatment initiation, as the titre may have risen in the interval between the drawing of the blood specimen and treatment initiation, which will make interpretation of any follow-up titre problematic.^82^ In the case of neurosyphilis or ocular syphilis, monitoring of clinical symptoms and serum RPR titres are considered sufficient, and routine repeat CSF analysis during follow-up is generally not required.

An adequate treatment response is defined as a reduction in the RPR titre of four-fold or greater that is expected within 1 year after treatment for early syphilis, and within 2 years after treatment for late latent syphilis.^83^ For instance, if the baseline RPR was 1:64, then an adequate response would be 1:16 or lower.

For early syphilis, it is recommended to perform an RPR at 6 months and 12 months post treatment, and at 6 months, 12 months and 24 months in late latent infection and tertiary disease to identify treatment failure early as evidenced by rising titre. It is important to note that the RPR titre does not always become negative with successful treatment – this phenomenon is known as a ‘serofast’ state and occurs in up to 50% of patients.^84^ As long as the titre has fallen by four-fold or greater, and preferably to 1:8 or lower, the response can be considered adequate. In case of a baseline titre of 1:4, 1:2 or 1:1, a stable titre post treatment is acceptable.

Rising RPR titres or failure of titres to decline appropriately at the end of the follow-up period should be managed by assessing the patient for a potential reinfection (treatment completion, partner treatment, sexual history with new partners) and the presence of neurosyphilis (Table 4). If these options are excluded, serology may be repeated once more 3–6 months later to check that the titre is not rising. If not, and the titre is 1:8 or lower, serofast state may be accepted, while further evaluation and treatment are required in cases with rising titres.

**TABLE 4:** Steps in the assessment of failure of the RPR titre to decline during follow-up after treatment.

Step	Activity	Interpretation
1.	Take history of treatment completion, sex partner(s) management, and potential new sexual partners.	If repeat infection is likely, retreat for early syphilis.
2.	Assess for symptoms and signs of neurosyphilis, ocular syphilis and otosyphilis.	If symptoms or signs are present, perform lumbar puncture to exclude neurosyphilis and treat accordingly if neurosyphilis is present.
3.	If a titre is rising (not just failing to decline) or remains high (> 1:8).	Perform lumbar puncture to exclude neurosyphilis even if the patient is asymptomatic. If present, treat accordingly. If excluded, treat for early syphilis.

## Public health response

Syphilis has serious health implications, and strengthening diagnosis and case management is essential to address the currently growing burden of infection. In line with the World Health Organization’s Global Health Sector Strategy,^85^ South Africa has committed to achieving the elimination of congenital syphilis targets by 2028.^86^ This requires the early (< 20 weeks gestational age) booking of pregnant women for ANC, screening regularly during pregnancy using RDTs, ensuring availability of BPG, and early initiation and high BPG treatment coverage as guided in the recently released Guideline for Vertical Transmission Prevention of Communicable Diseases by the South African National Department of Health.^65^

However, optimising screening and treatment of pregnant women alone will be unlikely to be sufficient to address the rising burden of infection in the country. Strengthening clinical case identification and management, and partner management is essential, and primary prevention, including the use of condoms and voluntary medical male circumcision, should be encouraged. In addition, scale-up of screening for asymptomatic infection in non-pregnant populations is recommended as part of comprehensive STI services in the Southern African HIV Clinicians Society’s guideline for management of STIs.^29^

## Conclusion

Syphilis is an ancient infection with multiple manifestations and health implications. This guideline provides recommendations on the recognition, diagnosis and management of the most common clinical presentations. The evidence base for syphilis management is limited, and specialist advice is recommended in the case of complicated clinical cases as well as when there are diagnostic difficulties or uncertainties.
